# Chronic Inflammation in Chronic Kidney Disease Progression: Role of Nrf2

**DOI:** 10.1016/j.ekir.2021.04.023

**Published:** 2021-05-04

**Authors:** Peter Stenvinkel, Glenn M. Chertow, Prasad Devarajan, Adeera Levin, Sharon P. Andreoli, Sripal Bangalore, Bradley A. Warady

**Affiliations:** 1 Department of Renal Medicine M99, Karolinska University Hospital at Huddinge, Karolinska Institutet, Stockholm, Sweden; 2 Division of Nephrology, Stanford University, Stanford, California, USA; 3 Department of Pediatrics, University of Cincinnati, Cincinnati, Ohio, USA; 4 Department of Medicine, The University of British Columbia, Vancouver, Canada; 5 Department of Pediatrics, Indiana University School of Medicine, Indiana University, Indianapolis, Indiana, USA; 6 Division of Cardiology, New York University, New York, New York, USA; 7 Division of Pediatric Nephrology, Children’s Mercy Kansas City, Kansas City, Missouri, USA

**Keywords:** chronic inflammation, chronic kidney disease, mitochondrial dysfunction, Nrf2, oxidative stress, resident kidney cells

## Abstract

Despite recent advances in the management of chronic kidney disease (CKD), morbidity and mortality rates in these patients remain high. Although pressure-mediated injury is a well-recognized mechanism of disease progression in CKD, emerging data indicate that an intermediate phenotype involving chronic inflammation, oxidative stress, hypoxia, senescence, and mitochondrial dysfunction plays a key role in the etiology, progression, and pathophysiology of CKD. A variety of factors promote chronic inflammation in CKD, including oxidative stress and the adoption of a proinflammatory phenotype by resident kidney cells. Regulation of proinflammatory and anti-inflammatory factors through NF-κB– and nuclear factor, erythroid 2 like 2 (Nrf2)–mediated gene transcription, respectively, plays a critical role in the glomerular and tubular cell response to kidney injury. Chronic inflammation contributes to the decline in glomerular filtration rate (GFR) in CKD. Whereas the role of chronic inflammation in diabetic kidney disease (DKD) has been well-elucidated, there is now substantial evidence indicating unresolved inflammatory processes lead to fibrosis and eventual end-stage kidney disease (ESKD) in several other diseases, such as Alport syndrome, autosomal-dominant polycystic kidney disease (ADPKD), IgA nephropathy (IgAN), and focal segmental glomerulosclerosis (FSGS). In this review, we aim to clarify the mechanisms of chronic inflammation in the pathophysiology and disease progression across the spectrum of kidney diseases, with a focus on Nrf2.

Pressure-mediated injury is a well-recognized mechanism for structural damage in CKD.^1^, ^2^, ^3^ Therapies that decrease intraglomerular pressure (angiotensin-converting enzyme inhibitors or angiotensin-receptor blockers) are frequently used for the treatment of CKD and have consistent effects on pathogenic mechanisms related to blood pressure and proteinuria. Nevertheless, the effects of angiotensin-converting enzyme inhibitors and angiotensin-receptor blockers on clinically meaningful outcomes, including slowing the progressive loss of kidney function and decreasing the incidence of ESKD, are more modest.^4^, ^5^, ^6^, ^7^, ^8^, ^9^ To attenuate, arrest, or reverse CKD progression, nephrologists will need to target pathogenetic mechanisms other than altered glomerular hemodynamics.^10^^,^^11^

Chronic inflammation and mitochondrial dysfunction are increasingly recognized as contributing to kidney fibrosis and ESKD.^12^^,^^13^ Regardless of CKD etiology, chronic inflammation is likely to be present as both a cause and a consequence of glomerular and tubulointerstitial pathology.^12^^,^^14^, ^15^, ^16^, ^17^ In many forms of CKD, proteinuria is a well-recognized predictor of disease progression^18^ and patients with asymptomatic proteinuria exhibit low-grade inflammation linked to endothelial dysfunction.^19^ Whereas proteinuria contributes to the pathology of CKD by inducing adverse changes in glomerular function, such as lack of selectivity of the glomerular barrier, glomerular hypertrophy,^20^ and direct damage to tubule epithelial cells, it also does so by promoting chronic inflammation.^21^^,^^22^

Results from a comprehensive pathway map analysis of gene sets linked to estimated GFR in 157 European patients with 9 different types of CKD indicated that inflammation and metabolism were the 2 main pathways in the pathology leading to CKD progression (Figure 1).^23^ Steady-state mRNA expression profiles across multiple etiologies of CKD revealed up-regulation of proinflammatory genes, including human leukocyte antigen isoforms, Toll-like receptors 1 and 3, and *NF-κB1*.^23^ This analysis revealed gene and protein interactions and defined known and novel mechanisms, which drive biological impairment across several kidney diseases, including thin basement membrane disease, FSGS, membranous nephropathy, minimal change disease, diabetic nephropathy, hypertensive nephropathy, IgAN, and lupus nephritis.^23^ Importantly, nuclear factor, erythroid 2 like 2 (Nrf2) anti-inflammatory pathway, discussed in detail subsequently in this review, served as a hub between 2 clusters of inflammatory and metabolic pathways activated across multiple etiologies of CKD, suggesting a common mechanism of inflammation and metabolism regulation.^23^ The aim of this comprehensive review is to discuss the role of inflammation and Nrf2 in the progression of CKD of different etiologies.

**Figure 1:**
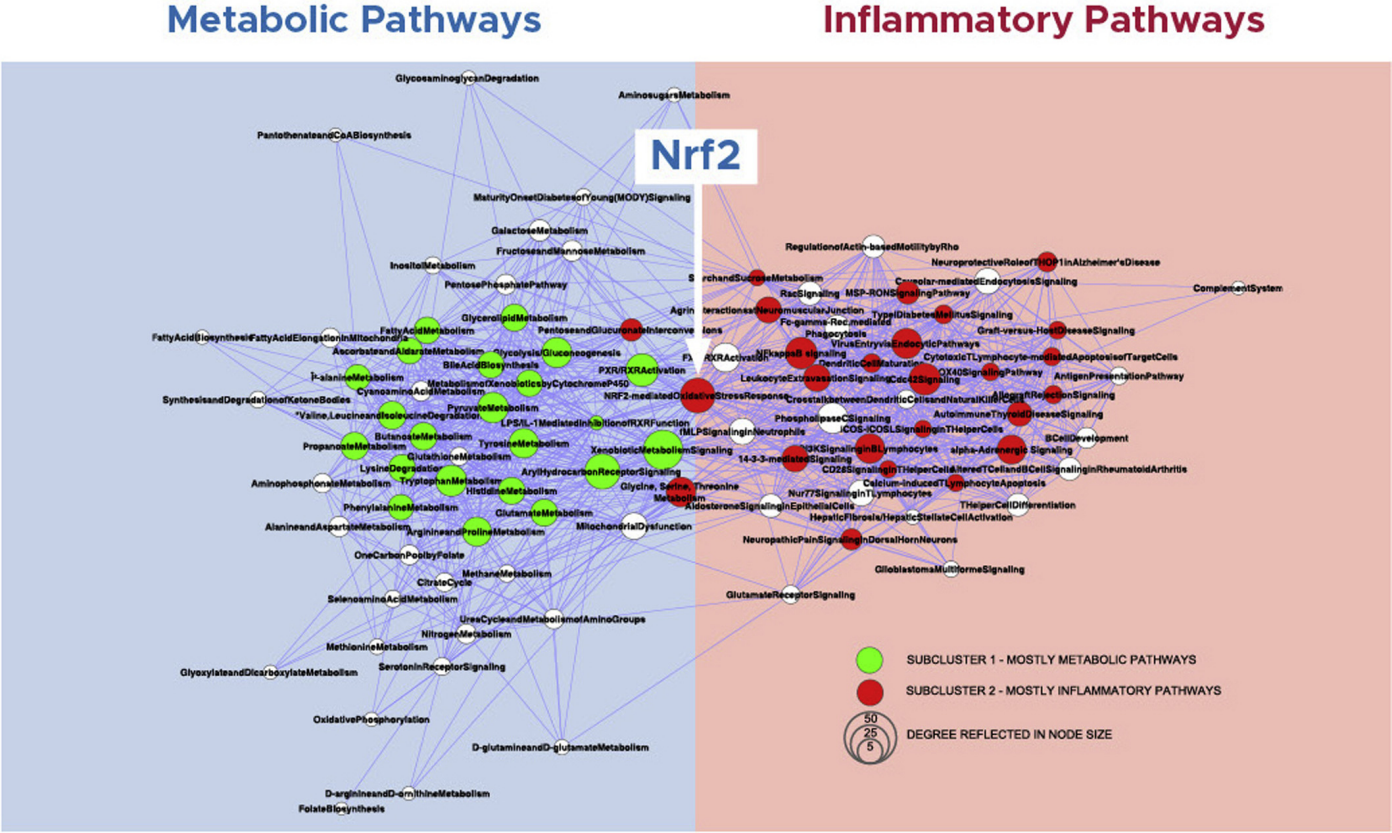
Inflammation and metabolism in CKD progression.^23^ Inflammation and metabolism are 2 main pathways leading to CKD progression, with Nrf2 serving as the hub. Reproduced with permission from the American Society of Nephrology, from: Integrative biology identifies shared transcriptional networks in CKD, Martini S *et al.*, Vol 25, Issue 11, copyright 2014; permission conveyed through Copyright Clearance Center, Inc. CKD, chronic kidney disease.

### Chronic Inflammation in the Progression of CKD

#### Chronic Inflammation and Oxidative Stress

Persistent low-grade inflammation^24^ and oxidative stress (i.e., elevation of reactive oxygen species [ROS])^25^ are partners in crime and common hallmarks of the uremic phenotype that promotes premature aging^26^ and kidney fibrosis.^27^^,^^28^ Inflammation and oxidative stress participate in a positive feedback loop, in which each amplifies the other.^29^ Oxidative stress induces inflammation by activating NF-κB with the subsequent production of cytokines (e.g., interleukin [IL]-1α, IL-1β, tumor necrosis factor [TNF], IL-6)^30^, ^31^, ^32^ associated with the progressive decline in estimated GFR.^33^ Data derived from the Chronic Renal Insufficiency Cohort study revealed that circulating IL-6 and TNF receptor 2 are associated with incident CKD^34^ and that TNF receptor 2 is independently associated with a more rapid loss of kidney function in CKD.^35^

#### Resident Kidney Cells in Chronic Inflammation

CKD progression occurs mainly by kidney fibrosis, a process in which activated myofibroblasts are the main collagen-producing cells.^36^, ^37^, ^38^ Collagen, predominantly fibrillar collagens I and III, is a major contributor to fibrosis-induced cellular loss in CKD.^39^ Among a number of approaches that reduce experimental fibrosis, such as targeting transcription factors, signaling and developmental pathways, and epigenetic modulators, such as microRNAs, anti-inflammatory approaches have received recent attention.^11^ As noted previously, chronic inflammation is closely linked to both CKD initiation and progression.^40^, ^41^, ^42^, ^43^ In contrast to acute inflammation, which is a natural immune response to kidney injury^44^^,^^45^ that plays a role in kidney tissue repair after exposure to harmful stimuli, chronic inflammation is a maladaptive response that results from persistent stimulation of proinflammatory signaling pathways.^2^^,^^3^^,^^17^^,^^44^^,^^46^^,^^47^

Although acute inflammation is marked by infiltrating white blood cells,^48^ chronic inflammation is characterized by the activation of resident kidney cells that exhibit a proinflammatory phenotype.^49^, ^50^, ^51^, ^52^, ^53^ Activation of resident kidney cells, including mesangial cells, endothelial cells, tubular epithelial cells, and podocytes, results in the production of proinflammatory chemokines responsible for perpetuating the cycle of chronic inflammation that eventually leads to kidney fibrosis and loss of kidney function (Figure 2).^50^^,^^53^^,^^54^

**Figure 2:**
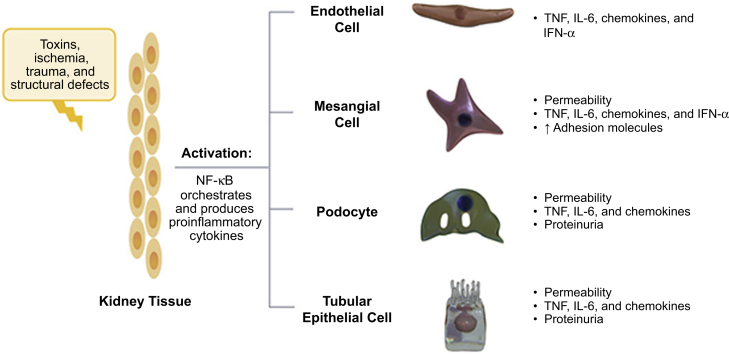
Activation of resident kidney cells contributes to chronic inflammation in CKD.^53^ Resident kidney cells proliferate and produce proinflammatory chemokines responsible for perpetuating the cycle of chronic inflammation leading to kidney fibrosis. Adapted by permission from Springer Nature. *Nat Rev Immunol*. The immune system and kidney disease: basic concepts and clinical implications. Kurts C *et al.* Copyright 2013. IFN-ɑ, interferon alpha; IL-6, interleukin 6; TNF, tumor necrosis factor.

Increased levels of inflammatory cytokines and deposition of extracellular matrix (ECM) causes tubulointerstitial fibrosis, mesangial expansion, and a subsequent decline in GFR.^55^ On activation, mesangial cells release chemokines and cytokines, which act locally on mesangial cells, other resident glomerular cells, and leukocytes. In the presence of chronic mesangial cell activation, ECM expansion in the interstitial space causes interstitial fibrosis, which leads to glomerulosclerosis.^50^ In the classic 5/6 nephrectomy CKD model, NF-κB is activated and transforming growth factor beta (TGF-β) is up-regulated.^56^ NF-κB is a regulator of proinflammatory genes that orchestrates and produces hundreds of inflammatory cytokines and mediators.^36^^,^^57^^,^^58^ Activation of TGF-β causes progressive fibrosis.^2^ Podocytes express TGF-β after the onset of proteinuria,^59^ which subsequently promotes the transformation of epithelial and mesangial cells into fibroblasts and myofibroblasts.^2^ TGF-β also causes podocytes to produce ECM proteins that accumulate in the tubulointerstitium.^59^ TGF-β is synthesized by tubular epithelial cells and myofibroblasts at various stages throughout the process of kidney fibrosis.^60^

#### Proinflammatory Mediators in CKD

Glomerular damage results from the failure to eradicate harmful proinflammatory stimuli in glomerular cells or genetic mutations leading to a proinflammatory state.^36^^,^^44^^,^^50^ Factors that may contribute to a proinflammatory tubular cell response include release of cytokines, leakage of albumin and complement proteins, hypoxia resulting from endothelial dysfunction, and direct injury owing to immunologic, infectious, toxic, metabolic, or ischemic insults.^17^ Moreover, senescence of tubular cells and podocytes promoting a senescence-associated secretory phenotype with increased local secretion of inflammatory proteins links loss of kidney function to tissue inflammation.^61^ Kidney hypoxia/ischemia contributes to progression of kidney disease by both inflammation and oxidative stress.^62^ Since Nrf2 deficiency enhances susceptibility to ischemia-reperfusion–induced kidney injury,^63^ up-regulation of Nrf2 may protect vulnerable kidneys against repeated episodes of ischemia during adverse clinical events.

Tubular cell activation promotes further damage through interstitial leukocyte recruitment and activation, secretion of profibrotic growth factors (e.g., platelet-derived growth factor, connective tissue growth factor, and TGF-β), and stimulation of myofibroblast accumulation and activation resulting in interstitial collagen deposition and fibrosis.^17^^,^^64^ The multiple inflammatory mediators involved in the complex processes of chronic inflammation, remodeling, fibrosis, and loss of kidney function are summarized in Figure 3.^36^ Genetic and epigenetic factors are strong determinants of inflammation in CKD.^23^^,^^65^ One extensive analysis indicated that genotype is correlated with the presence of inflammation in patients with CKD, as defined by circulating levels of high-sensitivity CRP, whereas phenotypic features less effectively distinguish patients with inflammation from those without.^66^

**Figure 3:**
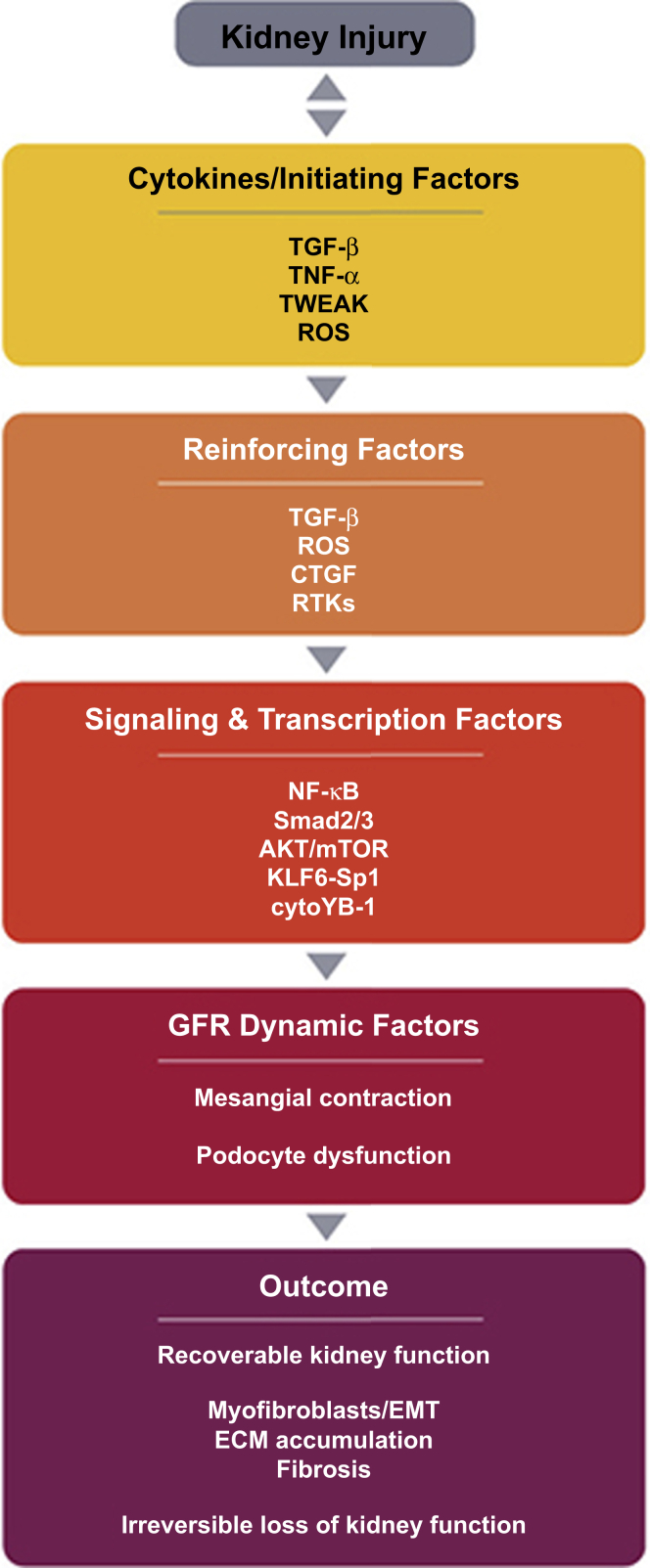
Mediators of chronic kidney disease.^36^ Various inflammatory mediators are involved in the complex processes leading to the loss of kidney function. Adapted with permission from Elsevier. Originally published in *Eur J Pharmacol*. Vol 820. Lv W *et al.* Inflammation and renal fibrosis: recent developments on key signaling molecules as potential therapeutic targets. Pages 65 to 76. Copyright 2018, Elsevier. ECM, extracellular matrix; EMT, epithelial-mesenchymal transdifferentiation; GFR, glomerular filtration rate; ROS, reactive oxygen species; TNF, tumor necrosis factor; TGF-β, transforming growth factor beta; TWEAK, TNF-like weak inducer of apoptosis.

### Proinflammatory Role of NF-κB in CKD

In CKD, the activation of ROS-generating enzymes (e.g., nicotinamide adenine dinucleotide phosphate oxidase and xanthine oxidase) contributes to elevated oxidative stress in the kidney.^67^ Oxidative stress triggers activation of NF-κB, enhancing the inflammatory response. NF-κB orchestrates and stimulates the production of a variety of inflammatory cytokines and mediators.^2^^,^^36^^,^^57^^,^^58^ Cytokines produced from many resident kidney cells recruit macrophages to damaged tissue, which results in increased production of proinflammatory factors and progressive glomerulosclerosis.^2^^,^^3^

### Anti-inflammatory Role of Nrf2 in CKD

Nrf2 represents a critical cellular factor that evolved as a protection against oxidative stress when organisms hundreds of millions of years ago began to explore the world above the oceans and were exposed to oxygen.^68^, ^69^, ^70^, ^71^ A member of the bZIP family of transcription factors—specifically in the CNC subfamily—Nrf2 has 7 structural domains (Neh1 to Neh7) with varying functions. Nrf2 heterodimerizes with sMaf proteins K, G, and F within the nucleus, which facilitates recognition of an enhancer sequence known as an antioxidant response element that is found in the regulatory regions of more than 250 genes.^3^^,^^70^^,^^72^, ^73^, ^74^

#### The Role of Nrf2 in Regulating Inflammation

Nrf2 protects kidney cells and other tissues by upregulating an array of genes and consequently attenuating the production of proinflammatory cytokines.^15^ Molecules with levels that are increased by Nrf2 include catalase, superoxide dismutase, glutathione peroxidase, heme oxygenase-1, reduced nicotinamide adenine dinucleotide phosphate quinone oxidoreductase, and glutamate-cysteine ligase.^15^ An equilibrium between protein synthesis and proteasomal degradation is required to maintain the intracellular concentration of Nrf2 at a low level.^74^ The cytosolic inhibitor Keap1 binds to Nrf2 in the cytoplasm.^67^^,^^71^^,^^75^

Under normal conditions, Keap1 targets Nrf2 for degradation by the ubiquitin-proteasome system (Supplementary Figure S1).^55^^,^^76^ Under conditions of oxidative stress, electrophiles and ROS alter the conformation of Keap1 by forming direct adducts with specific sensor cysteine residues.^55^^,^^76^ Such modifications alter the interaction between Keap1 and Nrf2, resulting in decreased Nrf2 degradation in cells exposed to oxidative stress. Nrf2 then translocates into the nucleus where it activates the transcription of its target genes.

Nrf2 also directly suppresses the expression of proinflammatory NF-κB target genes by binding to their promoters and inhibiting transcription.^77^ The substantial crosstalk between Nrf2 and NF-κB pathways controls the expression of multiple downstream target genes.^78^ Thus, the Keap1-Nrf2 system plays a key role in the resolution phase of inflammation by opposing oxidative damage and through inhibition of proinflammatory NF-κB signaling.^72^^,^^79^^,^^80^

Evidence from the animal kingdom suggests that Nrf2-based antioxidant defense mechanisms have evolved to protect species during extreme conditions.^68^ Inhibition of Nrf2 activity promotes stress-induced premature senescent phenotype^81^ and Nrf2 expression decreases with aging in mice.^82^ Based on recent findings showing that down-regulation of Nrf2 activity promoted oxidative stress and accelerated cellular senescence, it was suggested that drugs targeting Nrf2 signaling could suppress cellular senescence-associated pathologies.^83^ Since the activity of Nrf2 is reduced in Hutchinson-Gilford progeria syndrome, a rare syndrome of premature aging,^84^ and a study of Nrf2 knockout mice stressed by space travel showed increased generation of age-associated metabolites,^85^ Nrf2 may play a protective role in aging processes. Indeed, persistent low-grade inflammation (i.e., “inflammaging”) and decreased Nrf2 expression are prominent features of CKD and other burden-of-lifestyle diseases associated with premature aging.^68^ Kidney fibrosis and epithelial-mesenchymal transition—a process in which differentiated epithelial cells undergo a phenotypic conversion that generates matrix-producing fibroblasts and myofibroblasts—contribute to aging in the kidney^86^ and may reflect a premature aging process confined to the kidney tissue.^11^ As other organs undergo premature aging processes in the inflamed uremic milieu, progression of kidney disease due to fibrosis and inflammation often associate with the parallel development of a uremic phenotype characterized by vascular calcification, sarcopenia, osteoporosis, etc (Supplementary Figure S2).^87^

#### The Role of Nrf2 in Kidney Disease

Results from multiple studies highlight the critical role of Nrf2 in kidney disease.^88^, ^89^, ^90^, ^91^ A recent systematic review of 32 studies concludes that whereas Nrf2 expression was consistently downregulated in CKD, NQO1, and HO-1 showed varying alterations related to inflammation, comorbidities, and severity of kidney damage.^92^ Jiang *et al.*^69^ showed that kidney biopsy specimens from patients with DKD demonstrated high levels of glucose-induced ROS in mesangial cells as well as activation of Nrf2 and downstream genes. By immunohistochemistry, it was demonstrated that whereas Nrf2 was expressed at low levels in normal glomeruli, it was up-regulated in glomeruli from patients with DKD.^69^ In contrast, an analysis of 20 patients on hemodialysis, 20 additional patients with nondialysis-requiring CKD, and 11 healthy individuals that included evaluation of *Nrf2* and *NF-κB* expression with real-time polymerase chain reaction showed that *Nrf2* gene expression was reduced and *NF-κB* expression increased in peripheral blood mononuclear cells from patients on hemodialysis compared with those from healthy individuals and patients with nondialysis-requiring CKD.^93^

Animal experiments also demonstrate a key role for Nrf2 in controlling uremic inflammation. *Nrf2*-knockout mice administered streptozotocin have higher ROS production and more pronounced oxidative DNA damage and kidney injury vs similarly treated wild-type animals.^69^ Impaired Nrf2 activation has also been associated with kidney fibrosis and disease progression in a mouse model of focal glomerulosclerosis.^94^ In accordance, impaired Nrf2 signaling and NF-κB activation promote inflammation and oxidative stress in a mouse remnant kidney model.^15^^,^^95^

Nrf2 activation also prevents or attenuates fibrosis. Unilateral ureteral obstruction in mice results in the down-regulation of Keap1, allowing for the rapid accumulation of Nrf2 in the nucleus and induction of Nrf2-dependent gene expression, which prevents generation of ROS. However, longer-term obstruction leads to a progressive reduction in nuclear Nrf2 as well as reduced levels of antioxidants and increased oxidative stress, inflammation, fibrosis, and tubular damage.^96^ These studies reveal the potential variability of Nrf2 expression in CKD based on disease progression since Nrf2 may be up-regulated in early stages due to ROS but can be downregulated as the disease worsens and inflammation is exacerbated.

Sodium-glucose cotransporter 2 inhibitors—a new class of renoprotectors—have been found to promote a substantial reduction in albuminuria and a reduced risk of progression to ESKD in both type 2 diabetic and nondiabetic CKD.^97^ As part of the renoprotective effects of sodium-glucose cotransporter 2 inhibitors seems to be mediated by improvement of renal hypoxia^98^ accompanied with reduced inflammation^99^ and improvement of antioxidant defense expression,^100^ it is of interest that it was recently reported that dapagliflozin restrains apoptosis and activates autophagy in part by activation of Nrf2/HO-1 pathways.^101^

#### Mitochondrial Dysfunction and Nrf2 in the Progression of CKD

Accumulating evidence suggests that mitochondrial dysfunction promotes the development and progression of CKD irrespective of the underlying cause.^13^ Mitochondria are complex organelles with various functions, including the generation of adenosine triphosphate by oxidative phosphorylation.^102^

More recently, our understanding of the roles mitochondria play has expanded beyond adenosine triphosphate production to include an appreciation of their function as organelles that regulate cellular processes, such as proliferation, differentiation, and death.^103^ Mitochondria act as a central hub within the cell, sensing changes in the cellular milieu and rapidly redirecting metabolic intermediates to appropriately meet the demands placed on the cell.^104^ It is now apparent that metabolic reprogramming plays a critical role in the inflammatory response.^105^ Encountering a pathogen triggers a phenotypic switch in macrophages that is characterized by a decrease in the rate of oxidative phosphorylation and fatty acid oxidation and a robust increase in the rate of glycolysis, lipid synthesis, and ROS production.^106^^,^^107^ Although ROS are necessary to mount both an innate and an adaptive immune response against a variety of invaders, such as bacteria,^103^ their overproduction leads to oxidative stress resulting in tissue damage and dysfunction.^30^ This metabolic reprogramming is meant to be short-lived and restricted to the locale of infection or damage. Once the stress has been eliminated, it is critical that cells return to homeostasis—a balanced state where inflammatory processes are turned off, ROS are neutralized, and mitochondrial metabolism reverts to a normal state of oxidative phosphorylation.^108^ In CKD, however, this “off switch” fails and the damaging processes are sustained, ultimately leading to tissue damage and loss of kidney function.^44^^,^^73^

Nrf2 is known to reduce ROS levels and suppress inflammation; however, recent studies have found that this transcription factor also regulates cellular and mitochondrial metabolism.^30^^,^^109^, ^110^, ^111^, ^112^ Nrf2 directs metabolic reprogramming by regulating glucose and lipid metabolism, increasing efficient adenosine triphosphate production, promoting mitophagy, and increasing mitochondrial biogenesis.^112^^,^^113^ By suppressing inflammation, reducing ROS levels, and supporting the structural and functional integrity of mitochondria, Nrf2 improves the ability of the cell to recover from cellular stress and restores cellular homeostasis.^109^^,^^112^^,^^113^

The kidneys are highly metabolic organs that require large amounts of adenosine triphosphate to function normally.^102^ Owing to their high oxygen consumption, the kidneys are susceptible to damage caused by ROS, which can accelerate kidney disease progression.^13^^,^^114^ Overproduction of ROS and defective mitophagy, along with activation of apoptotic pathways, which are regulated by mitochondria, are interconnected drivers of CKD progression.^13^ Mitochondrial dysfunction in kidney cells seems to be implicated in the risk of kidney disease. Higher mitochondrial DNA copy number, a surrogate marker of mitochondrial function improvement, has been associated with a lower risk of CKD, independent of traditional risk factors and inflammation.^115^ In contrast, reduced expression of mitochondrial-derived peptides has been associated with inflammation and reduced expression of Nrf2 in CKD.^116^ Furthermore, mitochondrial dysfunction is believed to play a role in kidney fibrosis^117^ and the progression of kidney disease.^108^ Taken together, these findings suggest that these metabolic powerhouses may be a therapeutic target to attenuate progression of kidney disease.^118^ A recent study revealed that an analog of bardoxolone methyl, a potent activator of Nrf2, confers protection from proteinuria-induced mitochondrial damage to tubules both *in vitro* and in an animal model by improved mitochondrial redox balance and mitochondrial function.^21^

### Chronic Inflammation Across the Spectrum of Kidney Disease

#### Alport Syndrome

Chronic inflammation in Alport syndrome, similar to other conditions resulting in CKD, is a result of persistent activation of proinflammatory signaling pathways.^2^^,^^3^^,^^17^^,^^44^, ^45^, ^46^, ^47^ Activated macrophages have been found to contribute to disease progression in Alport syndrome.^2^ *COL4A3* knockout mice develop proteinuria as early as 5.5 weeks of age.^119^ Excessive protein in the glomerular filtrate (“pathological proteinuria”) activates proinflammatory and profibrotic signaling pathways in proximal tubular epithelial cells^2^ and leads to the expression of genes encoding chemotactic molecules. These molecules promote infiltration of immune cells, leading to tubular atrophy and tubulointerstitial fibrosis.^2^ TGF-β is also involved in the processes of glomerulopathy and fibrosis.^2^^,^^59^^,^^120^^,^^121^ As patients with Alport syndrome experience a loss of kidney function despite the use of angiotensin-converting enzyme inhibitors and angiotensin-receptor blockers and there is a documented inflammatory component, the effects of Nrf2 stimulation with bardoxolone methyl are currently investigated in a randomized controlled trial (CARDINAL).^122^

#### Autosomal-Dominant Polycystic Kidney Disease

Inflammation is evident early during the course of ADPKD when patients have normal or near-normal kidney function. Results from several studies support a role for inflammation in ADPKD cyst development and disease progression. Cyst formation has been found to precede interstitial macrophage accumulation suggesting these cells are migrating to sites of inflammation.^123^^,^^124^ Regulation of cyst growth by macrophage migration inhibitory factor provides further support for inflammation as a stimulating factor in cyst development and for the involvement of macrophages in ADPKD.^125^ Animal studies also support a significant role for inflammation in the development and progression of ADPKD. For example, macrophage depletion in polycystin 1, a transient receptor potential channel interacting (*Pkd*) 1-targeted PKD mouse, resulted in a less severe cystic phenotype and better kidney function.^126^ NF-κB has been detected in the nuclei of cyst-lining cells in a mouse PKD model,^127^ and elevated levels of proinflammatory cytokines, including IL-1β, TNF, and IL-2, have been reported in ADPKD.^128^^,^^129^ These observations indicate that chronic inflammation may not only promote the initiation of disease and cystogenesis through its cellular effectors but also play a role in cyst expansion and disease progression.^123^^,^^125^ Indeed, it was recently reported that activation of Nrf2 ameliorates oxidative stress and cystogenesis in a mouse model of ADPKD.^91^

#### IgA Nephropathy

Inflammation in IgAN results from immune complex formation of galactose-deficient IgA1 in the glomerular mesangium.^130^ Immune complex formation arises subsequent to multiple sequential immunopathogenic “hits,”^131^, ^132^, ^133^ including induction of local inflammatory responses, IgA deposition in glomeruli, and activation of—and damage to—mesangial cells. Activated mesangial cells secrete components of ECM and release multiple mediators that contribute to kidney injury, including proinflammatory and profibrotic cytokines,^134^ which stimulate mesangial cell proliferation and recruitment of inflammatory cells into the glomerulus.^132^ Inflammatory mediators also modify gene expression in podocytes, resulting in podocyte injury (“glomerulopodocytic crosstalk”) and filtration of IgA immune complexes, including segmental glomerulosclerosis.^132^^,^^135^, ^136^, ^137^, ^138^ An animal study has suggested that stimulation of the Nrf2 pathway has the potential to modulate inflammation in IgAN. In mice with induced accelerated and progressive IgAN, stimulation of the Nrf2 pathway with antroquinonol inhibited T cell activation and prevented activation of the NLR family pyrin domain containing 3 inflammasome. It also significantly improved proteinuria, kidney function, and histopathology in accelerated and progressive-IgAN mice with established disease.^139^

#### Diabetic CKD

Proinflammatory signaling pathways and their downstream products are emerging as new biomarkers in DKD and may be promising therapeutic targets in patients with this disease.^140^ DKD involves activation of chronic inflammatory pathways that contribute to disease progression^141^ and is associated with multiple inflammatory cell types, molecules, and pathways, including macrophages, mast cells, and NF-κB–mediated transcription of inflammatory cytokines, including IL-1, IL-6, IL-18, and TNF.^141^, ^142^, ^143^, ^144^, ^145^ A signature of circulating inflammatory proteins enriched in the TNF receptor superfamily members predicted the 10-year risk of ESKD in diabetes,^146^ suggesting drugs targeting inflammation could help arrest progression of DKD. Ultrastructural changes in the glomerular basement membrane result from the presence of acute-phase markers of inflammation, such as IL-6.^147^ NF-κB–induced molecules and pathways result in structural alterations and functional abnormalities characteristic of DKD, and ultimately, kidney failure in these patients.^141^ A role for Nrf2 in DKD was found in a study of diabetic rats revealing impaired kidney function was correlated with oxidative stress and reduced translocation of Nrf2 into the nucleus.^148^ Results from a study of streptozotocin-treated, *Nrf2*-knockout mice revealed reduced protection against inflammation, impaired kidney function, fibrosis, and oxidative damage.^149^

#### Focal Segmental Glomerulosclerosis

Although podocyte injury and loss may be the primary drivers of FSGS,^150^ inflammation is also thought to play a key role in disease progression.^151^^,^^152^ Involvement of inflammation in the etiology of FSGS was suggested by the finding of higher interstitial cluster of differentiation 3-positive T cells and macrophages in kidney biopsies from patients with FSGS.^153^ Oxidative stress also contributes to the pathogenesis of FSGS.^154^ Damage to podocytes leads to further injury mediated by cytokine release (e.g., TGF-β), which results in the recruitment of monocytes, macrophages, and T cells and enhanced expression and secretion of other cytokines (e.g., IL-1 and TNF) and chemokines.^152^ Baseline kidney function in patients with FSGS is inversely correlated with the extent of global sclerosis and tubulointerstitial fibrosis, including urinary excretion of IL-12, interferon-γ, IL-4, IL-5, and IL-13.^155^ The infiltration of inflammatory cells in FSGS results in the accumulation of mesangial ECM, which can cause glomerular collapse (“collapsing variant”).^152^ Damage to tubular epithelial cells leads to a transformation to mesenchymal cells, resulting in collagen matrix deposition and tubulointerstitial fibrosis.^152^^,^^156^ This proinflammatory and profibrotic milieu has been involved in the progression of FSGS and may cause glomerular scarring and eventual ESKD.^157^ A role for Nrf2 in the progression of FSGS was found in the Imai rat model, in which impaired Nrf2 signaling in conjunction with NF-κB activation promoted inflammation and oxidative stress, both of which were associated with progressive glomerulosclerosis.^158^

### Conclusion

Mounting evidence suggests a significant role of inflammation and metabolic pathways in the progression of CKD of multiple etiologies.^23^ Pathway-crosstalk analysis of gene sets linked to estimated GFR reveals that most CKD signaling pathways aggregate in either an inflammation- or a metabolism-related cluster.^23^ Steady-state mRNA expression patterns across the spectrum of CKD are consistent with up-regulation of inflammatory genes.^23^ The Nrf2 pathway serves as a hub linking metabolic and inflammatory pathways in CKD.^23^ Repressed expression of this cytoprotective factor is linked to several pathogenic mechanisms known to promote fibrosis and progression of kidney disease, such as senescence, inflammation, mitochondrial dysfunction, and tissue hypoxia; therefore, Nrf2 activation is an attractive target for arresting progression of CKD.^12^^,^^13^^,^^61^^,^^62^ Augmenting the action of Nrf2 and its downstream mediators in CKD has the potential to attenuate, arrest, or even reverse the decline in kidney function.

## Disclosure

PS: scientific advisory boards for Reata Pharmaceuticals, Inc., AstraZeneca, Vifor Pharma, and Baxter Healthcare. GMC: board of directors for Satellite Healthcare, Inc.; consultant/advisor for Akebia Therapeutics, Amgen, Ardelyx, Inc., AstraZeneca, Baxter Healthcare, CloudCath, Cricket Health, DiaMedica Therapeutics, Inc., Durect Corp, DxNow, Inc., Gilead Sciences, Inc., Miromatrix Medical, Inc., Outset Medical, Reata Pharmaceuticals, Inc., Sanifit, and Vertex Pharmaceuticals Inc. PD: medical advisory board for Reata Pharmaceuticals, Inc.; speakers bureau for BioPorto Inc.; key opinion leader for Alnylam Pharmaceuticals and Dicerna Pharmaceuticals; coinventor on submitted patents for the use of NGAL as a biomarker of kidney injury; licensing agreements with Abbott Diagnostics and BioPorto Inc. for the development of NGAL as a biomarker of kidney injury. AL: advisory committee for Reata Pharmaceuticals, Inc.; grants from Otsuka America Pharmaceutical, Inc., AstraZeneca, and Boehringer Ingelheim International GmbH. SPA: consultant advisor to Alexion Pharmaceuticals and Reata Pharmaceuticals, Inc. SB: advisory committee for Reata Pharmaceuticals, Inc. BAW: advisory committee for Reata Pharmaceuticals, Inc.; medical advisory committee of the Alport Syndrome Foundation; consultant for Bayer AG, Akebia Therapeutics, Relypsa, Inc., Amgen, FibroGen, Inc., and UpToDate, Inc.; research support from the National Institutes of Health and Baxter Healthcare.

## Supplementary Material

Supplementary File (PDF)

**Figure S1.** Molecular mechanism of the Keap1-Nrf2 system during the oxidative stress response^76^ Under normal conditions, Nrf2 is degraded and inactivated after being captured by Keap1 homodimers. Under conditions of oxidative stress, the interaction between Keap1 and Nrf2 is inactivated, resulting in decreased Nrf2 degradation. Nuclear translocation of stabilized Nrf2 allows for transcriptional activation of Nrf2 target genes. Cys, cysteine residues; Keap1, kelch-like ECH-associated protein-1; Nrf2, nuclear factor, erythroid 2 like 2; Ub, ubiquitin-proteasome–dependent degradation. Reproduced with permission from Nezu M et al. Targeting the KEAP1-NRF2 system to prevent kidney disease progression. *Am J Nephrol*. 2017;45(6):473-483. Copyright © 2017 Karger Publishers, Basel, Switzerland.

**Figure S2.** Progression of kidney disease as part of premature aging processes in CKD.^87^ The toxic uremic milieu promotes inflammation and repressed expression of Nrf2, a phenomenon linked to oxidative stress, mitochondrial dysfunction, tissue hypoxia, and senescence. Evidence suggests that these features are major drivers of a prematurely aged phenotype in CKD, including early vascular aging (vascular calcification), sarcopenia, osteoporosis, heart failure, depression, and cognitive dysfunction. In addition, the same features may drive early aging in the kidney by kidney fibrosis and inflammation. In addition to hemodynamic and metabolic factors, inflammation and fibrosis may drive progression of kidney disease, creating a vicious circle. Nephrologists may intervene in this scenario by using established, novel, and putative future treatment strategies, such as ACEi/ARB, SGLT2i, Nrf2 agonist, and MR inhibitors. Angiotensin-converting enzyme inhibitor/angiotensin-receptor blocker (ACEi/ARB); CKD, chronic kidney disease; MR, mineralocorticoid receptor; Nrf2, nuclear factor, erythroid 2 like 2; sodium-glucose transport protein 2 inhibitor (SGLT2i).
