# Supplementary material for: Chronic Inflammation in Chronic Kidney Disease Progression: Role of Nrf2
Source: Kidney Int Rep. 2021 May 4;6(7):1775–87. doi: 10.1016/j.ekir.2021.04.023 (PMC8258499; doi:10.1016/j.ekir.2021.04.023)
Supplement: Supplementary File (PDF) [file mmc1.pdf]

Supplementary Figures

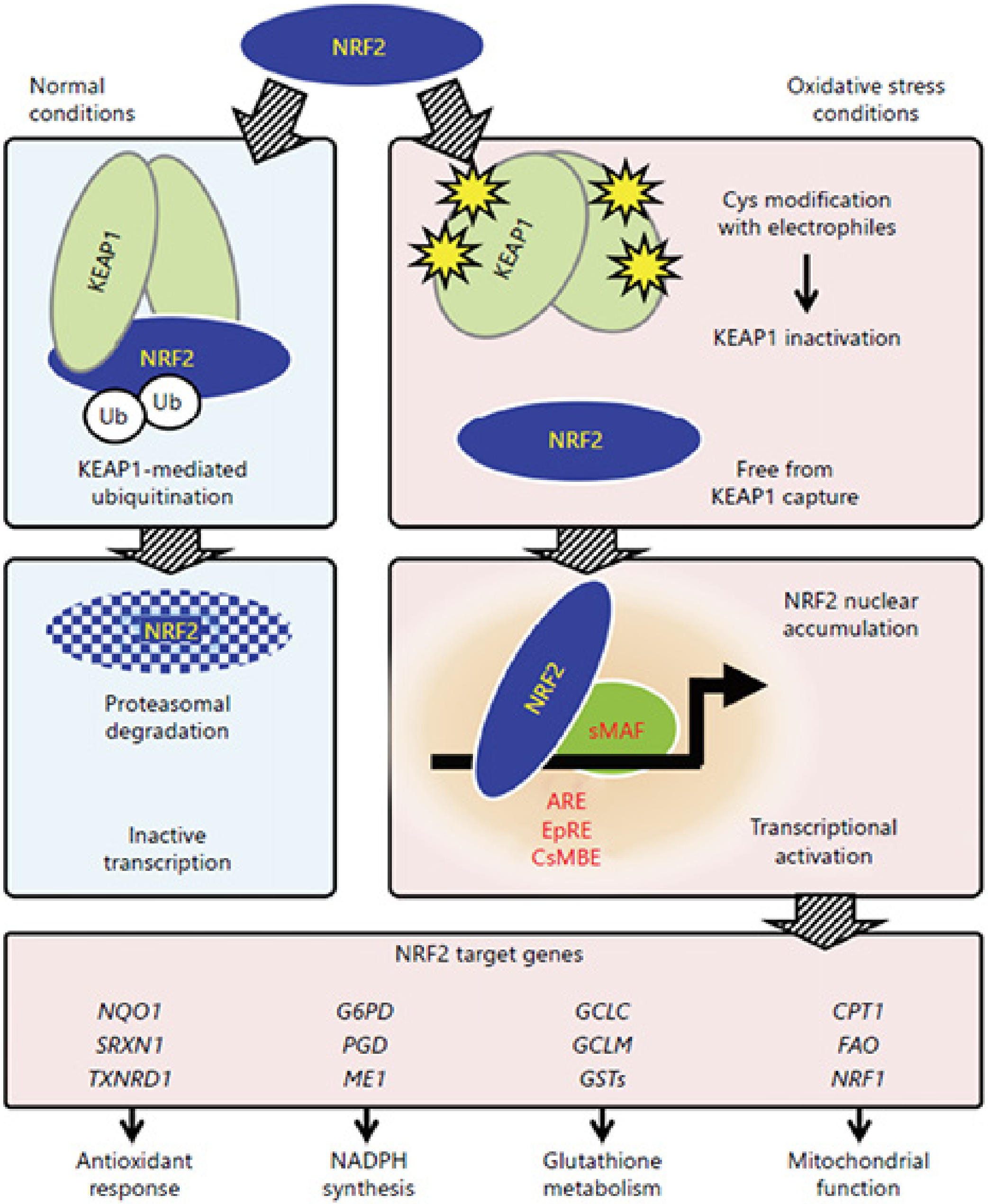

Figure S1. Molecular mechanism of the Keap1-Nrf2 system during the oxidative stress response[76] Under normal conditions, Nrf2 is degraded and inactivated after being captured by Keap1 homodimers. Under conditions of oxidative stress, the interaction between Keap1 and Nrf2 is inactivated, resulting in decreased Nrf2 degradation. Nuclear translocation of stabilized Nrf2 allows for transcriptional activation

of Nrf2 target genes. Cys, cysteine residues; Keap1, kelch-like ECH-associated protein-1; Nrf2, nuclear factor, erythroid 2 like 2; Ub, ubiquitin-proteasome–dependent degradation. Reproduced with permission from Nezu M et al. Targeting the KEAP1-NRF2 system to prevent kidney disease progression. *Am J Nephrol*. 2017;45(6):473-483. Copyright © 2017 Karger Publishers, Basel, Switzerland.

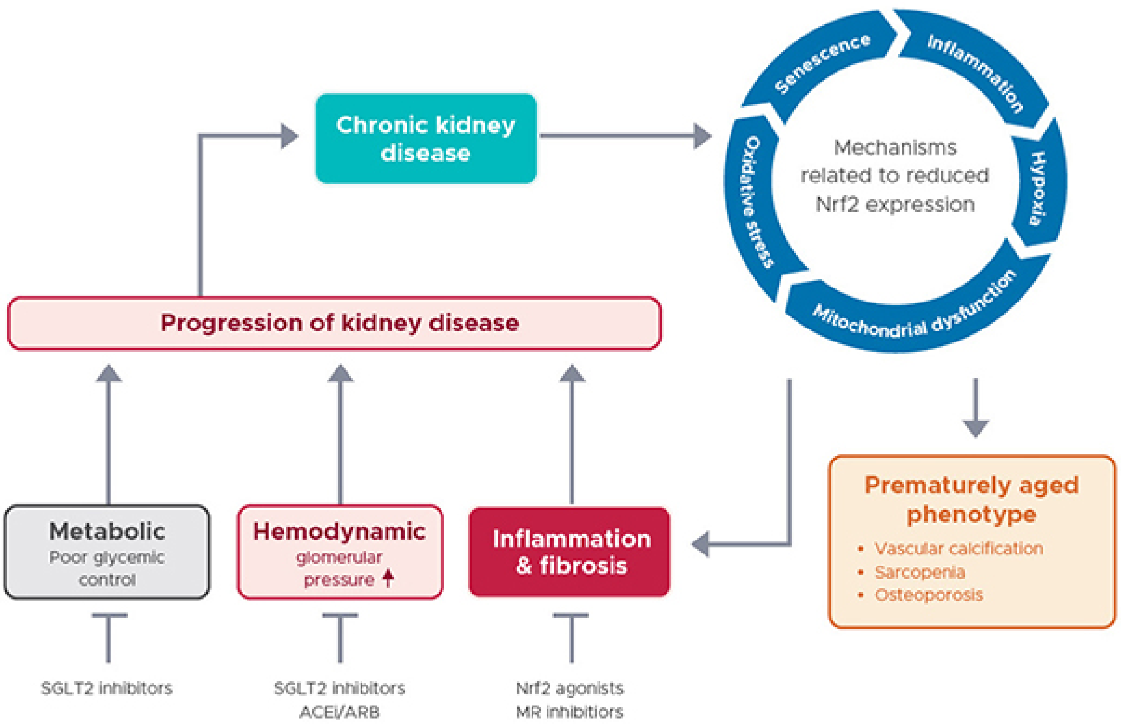

Figure S2. Progression of kidney disease as part of premature aging processes in CKD.[87] The toxic uremic milieu promotes inflammation and repressed expression of Nrf2, a phenomenon linked to oxidative stress, mitochondrial dysfunction, tissue hypoxia, and senescence. Evidence suggests that these features are major drivers of a prematurely aged phenotype in CKD, including early vascular aging (vascular calcification), sarcopenia, osteoporosis, heart failure, depression, and cognitive dysfunction. In addition, the same features may drive early aging in the kidney by kidney fibrosis and inflammation. In addition to hemodynamic and metabolic factors, inflammation and fibrosis may drive progression of kidney disease, creating a vicious circle. Nephrologists may intervene in this scenario by using established, novel, and

putative future treatment strategies, such as ACEi/ARB, SGLT2i, Nrf2 agonist, and MR inhibitors. Angiotensin-converting enzyme inhibitor/angiotensin-receptor blocker (ACEi/ARB); CKD, chronic kidney disease; MR, mineralocorticoid receptor; Nrf2, nuclear factor, erythroid 2 like 2; sodium-glucose transport protein 2 inhibitor (SGLT2i).
